# A Novel Asymmetric High-Performance MEMS Pendulum Capacitive Accelerometer

**DOI:** 10.3390/mi16101122

**Published:** 2025-09-30

**Authors:** Guangxian Dong, Jia Jiang, Weixin Wu, Zhentao Zhang, Jin Cao, Zhang Gao, Haitao Liu

**Affiliations:** 1 Chongqing Academy of Metrology and Quality Inspection, Chongqing 401121, Chinam13808326176@163.com (Z.G.); 2 24th Research Institute of China Electronics Technology Group Corporation, Chongqing 401332, China; 3 Key Laboratory of Fundamental Science of Micro/Nano-Device and System Technology, Chongqing University, Chongqing 400044, China

**Keywords:** asymmetric, MEMS (microelectromechanical system), pendulum, capacitive accelerometer, interface circuits

## Abstract

In this study, we propose a novel asymmetric high-performance MEMS pendulum accelerometer comprising a sensitive structure and an interface circuit. The sensitive structure, designed with asymmetric mass blocks, significantly improves both sensitivity and structural stability. The sensor is fabricated using a double-side polished (100) N-type silicon wafer and its structure is ultimately realized through ICP (Inductively Coupled Plasma) etching. We also develop and fabricate the corresponding interface circuit. The accelerometer is evaluated through a static field roll-over test, demonstrating excellent performance with a sensitivity of 1.247 V/g and a nonlinearity of 0.8% within the measurement range of −2 g to 2 g.

## 1. Introduction

Accelerometers are applied in various industries, including in automotive electronics (e.g., in airbag triggering systems), in vibration monitoring of industrial equipment, and for aerospace attitude control and inertial navigation [1,2,3]. Their widespread use results in significant demand for accelerometers with high accuracy and reliability [4,5]. Among different types of accelerometers, pendulum accelerometers are used extensively to measure high-performance acceleration signals, particularly in challenging contexts, such as oil field drilling, due to their simple structure, established technological foundation, high reliability, and strong anti-interference capability [6,7].

The performance of a pendulum accelerometer is primarily determined by the mechanical properties of the pendulum [8,9,10], the design of the control system [11,12], and characteristics of elastic deformation. In the context of mature mechanical and circuit architectures, a well-designed sensitive unit and interface circuit play a crucial role in achieving optimal performance [13,14]. In this paper, we propose a novel asymmetric high-performance MEMS pendulum capacitive accelerometer. The working principle and structural design are first introduced, followed by a detailed description of the sensor and interface circuit design and fabrication. Subsequently, experimental results for both the circuits and the accelerometer are presented, leading to the final conclusions.

## 2. Principle and Structure

The pendulum MEMS capacitive accelerometer consists of an MEMS sensitive structure and an interface circuit. When the system receives an acceleration input, the sensitive structure will produce a certain rotational displacement under the action of inertial torque. The change in rotational displacement is converted into a change in capacitance. The interface circuit detects and processes the capacitance change signal and ultimately obtains an output signal that can represent the magnitude of the acceleration signal, thus detecting the acceleration signal.

**Figure 1:**
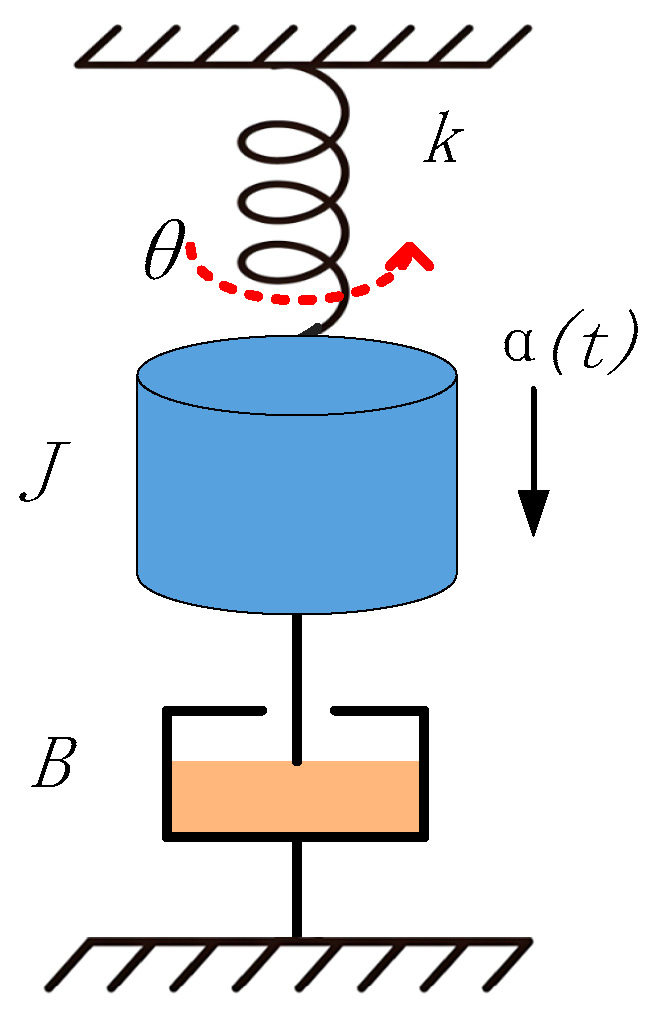
Mechanical model of pendulum accelerometer.

The sensitive structure of the pendulum MEMS capacitive accelerometer can be approximated as a second-order system representing the mass–elastic–structure damping characteristics of a torsion pendulum [15], as shown in Figure 1.

Under the an acceleration *α*(*t*) load, due to the effect of inertial torque, the sensitive structure will rotate around a fixed axis, and the rotation angle *θ*(*t*) satisfies the following dynamic Equation (1):

(1) $$J\frac{d^{2}\theta (t)}{dt^{2}}+B\frac{d\theta }{dt}+k\theta \left(t\right)=K_{a}\alpha (t)$$

where *J* is the moment of inertia; *B* is viscous damping; *k* is the rotational elastic modulus; *K_a_* is the torsion coefficient; and *α*(*t*) is the input acceleration.

The relationship between the swing angle *θ* of the sensitive mass and the acceleration a can be obtained by Equation (2):

(2) $$\theta =\frac{K_{a}/J}{\omega_{n}^{2}}\alpha =\frac{K_{a}}{k}\alpha$$

where *ω_n_* is the resonant frequency, $\omega_{n}=\sqrt{k/J}$.

**Figure 2:**
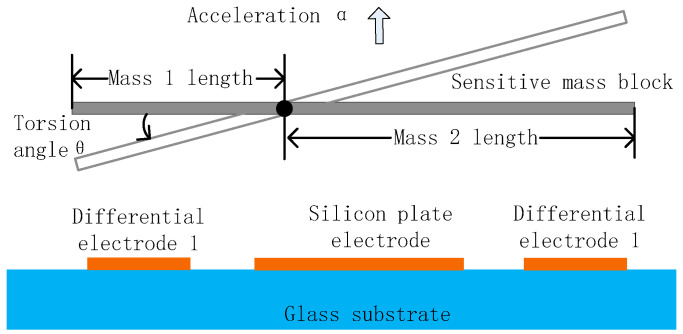
Simplified structure diagram of pendulum accelerometer.

The simplified structure of the pendulum accelerometer primarily consists of a sensitive mass block, a torsion beam, a fixed end, a glass substrate, and a signal detection circuit, as shown in Figure 2. When the acceleration sensor is subjected to vertical external acceleration acting on the sensitive mass block, the differences in inertia moments and masses between the left and right sensitive mass blocks relative to the torsion beam induce torsional displacement around the beam. Simultaneously, a differential detection capacitor is formed between the mass block and the glass substrate plate, enabling the conversion of the variation in torsional displacement into a corresponding change in capacitance. The signal detection circuit is capable of measuring this capacitance variation and converting it into a detectable voltage signal, thereby enabling the indirect determination of the pendulum accelerometer’s acceleration magnitude and, ultimately, detecting acceleration.

The resulting change in differential detection capacitance is formulated in Equations (3) and (4):

(3) $$C_{1}=\frac{\varepsilon_{0}\varepsilon A}{d_{0}-\Delta d}=\frac{\varepsilon_{0}\varepsilon A}{d_{0}}\left(\frac{1}{1-\Delta d/d_{0}}\right)=C_{0}\left(\frac{1}{1-\Delta d/d_{0}}\right)$$

(4) $$C_{2}=\frac{\varepsilon_{0}\varepsilon A}{d_{0}+\Delta d}=\frac{\varepsilon_{0}\varepsilon A}{d_{0}}\left(\frac{1}{1+\Delta d/d_{0}}\right)=C_{0}\left(\frac{1}{1+\Delta d/d_{0}}\right)$$

where *ε*_0_ is the dielectric constant of vacuum, *ε* is the relative dielectric constant between the sensitive mass and the glass substrate medium, A is the relative area between the sensitive mass and the glass substrate, and *d*_0_ is the initial electrode spacing between the sensitive mass and the differential detection electrode.

From the Taylor expansion of Equations (3) and (4) and when Δ*d* < *d*_0_, the capacitance change of the differential detection electrode at this time can be obtained as Equation (5):

(5) $$∆C\approx 2C_{0}\frac{∆d}{d_{0}}=2C_{0}\frac{L\theta }{d_{0}}$$

where *L* is the distance from the center of the differential detection electrode to the center of the rotating shaft, and *C*_0_ is the static capacitance.

By combining Equations (2) and (5), the relationship between the capacitance change and the input acceleration can be obtained as Equation (6):

(6) $$a=\frac{k\theta }{K_{a}}=\frac{kd_{0}\Delta C}{2C_{0}LK_{a}}$$

From the above equation, it can be concluded that the change in capacitance is directly proportional to the input acceleration, and the change in capacitance can be detected to calculate the magnitude of the system’s acceleration.

## 3. Design and Simulations

### 3.1. Sensor Sensitive Unit

**Figure 3:**
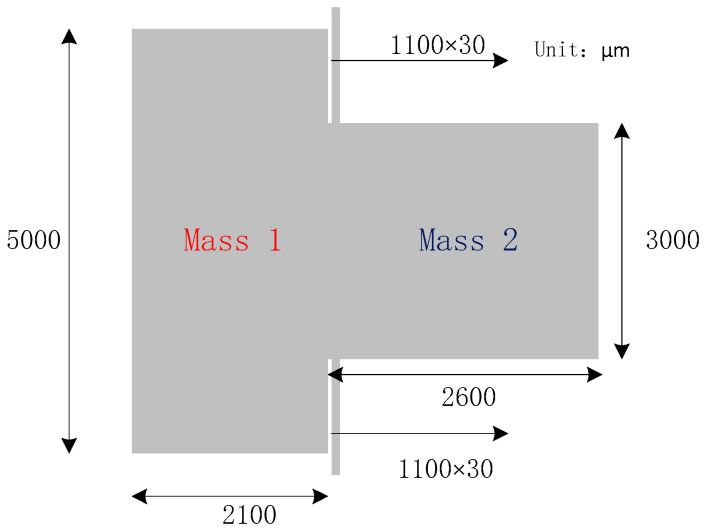
Structural design diagram of pendulum accelerometer.

The sensitive element pendulum accelerometer is designed using an asymmetric mass block; the structure diagram is shown in Figure 3. The structural parameters are shown in Table 1, and the material properties of the pendulum accelerometer are shown in Table 2.

**Figure 4:**
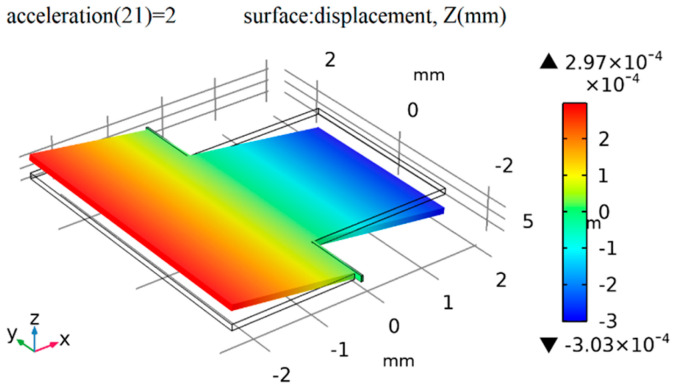
Displacement contour under +2 g of acceleration.

Figure 4 shows the results of the static force analysis of the sensor; the color scale bar indicates the displacement. The simulation analysis results show that the displacement of the proof mass reaches its maximum value of 0.303 μm when there is 2 g of acceleration along the z-axis, so the mechanical sensitivity is 0.152 μm/g.

**Figure 5:**
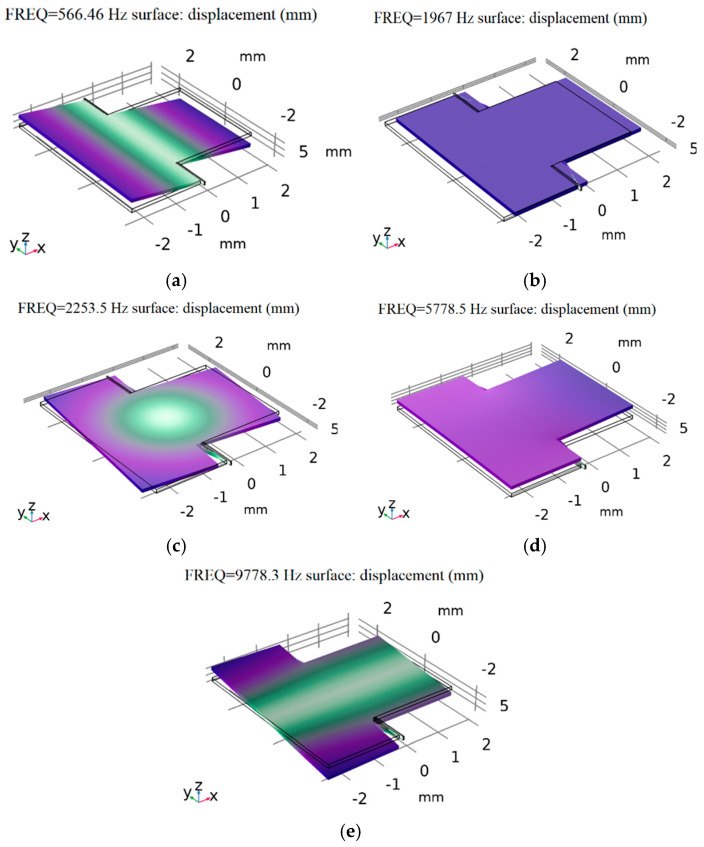
Modal graph of the pendulum accelerometer: (**a**) first, (**b**) second, (**c**) third, (**d**) fourth, and (**e**) fifth modes.

The resonant frequency and modal response of the sensor were analyzed using finite element simulation (FEM) analysis. The results obtained via COMSOL Multiphysics 5.5, are shown in Figure 5 and Table 3. Figure 5a shows the first working mode of the accelerometer; the resonant frequency is 566.46 Hz. Figure 5b shows the second mode, in which the proof mass translates along the x-axis and the resonant frequency is 1967 Hz. Figure 5c shows the third mode, in which the proof mass rotates around the z-axis, and the resonant frequency is 2253.5 Hz. Figure 5d shows the fourth mode, in which the support beams translate along the z-axis, and the resonant frequency is 5778.5 Hz. Figure 5e shows the fifth mode, in which the proof mass rotates around the x-axis. The interfering modes were far from the operating mode.

**Figure 6:**
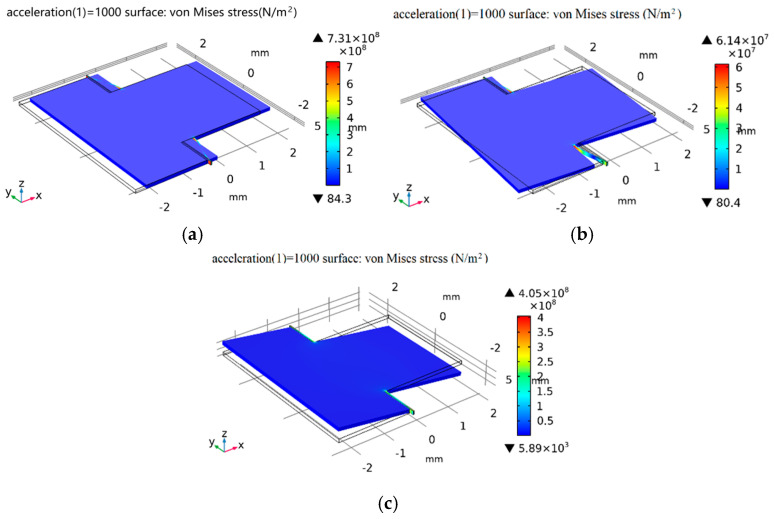
Stress plots under 1000 g shock acceleration in (**a**) x-axis, (**b**) y-axis, and (**c**) z-axis directions.

We applied an impact acceleration of 1000 g in the x, y, and z directions to conduct a strength stress simulation analysis on the structure; the stress results are shown in Figure 6, in which the color scale bar indicates the displacement. It can be observed that the maximum stress values in the x-, y-, and z-axis directions are 731 MPa, 61.4 MPa, and 405 MPa, respectively, and the ultimate stress value is far lower than that of the silicon material.

### 3.2. Damping Analysis

**Figure 7:**
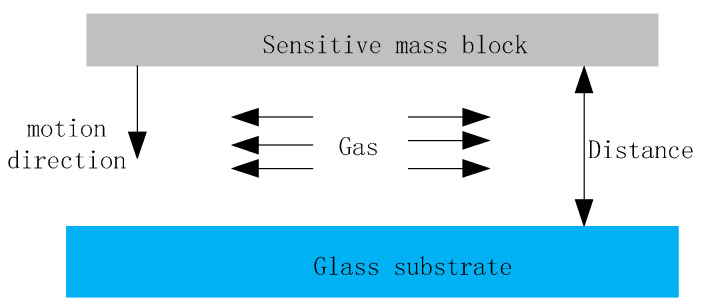
Schematic diagram of squeeze film damping.

In the structure of the MEMS pendulum accelerometer, the influence of slide film damping is significantly smaller than the influence of squeeze film damping; therefore, the damping effect is generated when the release structure moves and the gap between the sensitive mass block and the glass substrate increases or decreases. When the gap becomes smaller, the gas film between the two surfaces will be compressed, resulting in a damping force, as shown in Figure 7.

Using the theory of fluid mechanics, a model was established based on the Reynolds equation to describe the damping generated by the compressed gas film [16]. The compression coefficient of the gas film in the electrode spacing is defined as in Equation (7):

(7) $$\sigma =\frac{12\mu l^{2}}{P{d_{0}}^{2}}\omega$$

where *μ* is the gas viscosity coefficient, *l* is the length of the sensitive mass block, *P* is the gas pressure, *d*_0_ is the distance between the proof mass and the differential detection electrode, and *ω* is the resonant frequency.

### 3.3. Process and Fabrication

**Figure 8:**
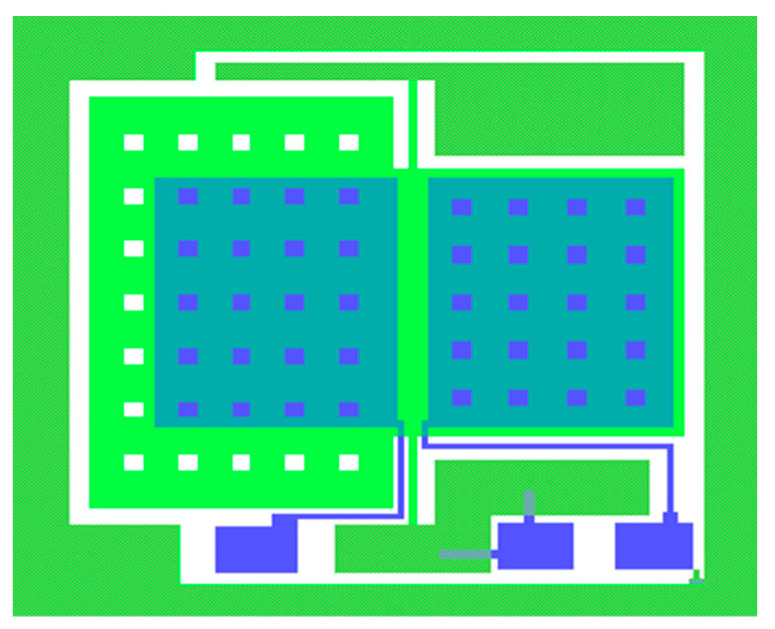
Layout of the pendulum accelerometer.

The sensor consists of three layers: a glass gold electrode layer, a bonding anchor layer, and an etching structure layer. The total layout of the sensitive structure unit is shown in Figure 8. To reduce the impact of membrane damping on the structure, square damping holes are etched on the mass block to reduce the air damping between the plates. The side length of the damping holes is 120 μm, and it is estimated that this will cause a capacitance reduction of approximately 4.3%, which is relatively small compared to the overall capacitance.

**Figure 9:**
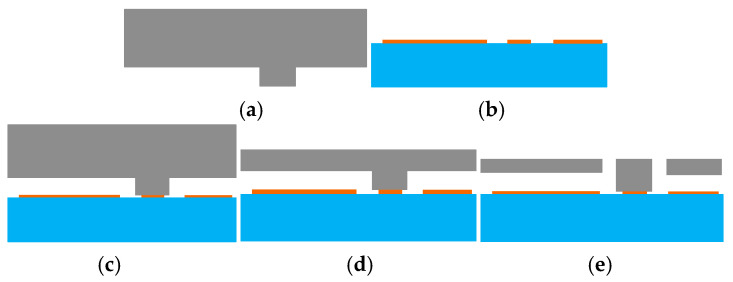
Fabrication of MEMS pendulum accelerometer: (**a**) corrosion of silicon, (**b**) growth of electrodes on glass, (**c**) bonding silicon and glass, (**d**) thinning silicon, and (**e**) ICP structure release to form beam.

**Figure 10:**
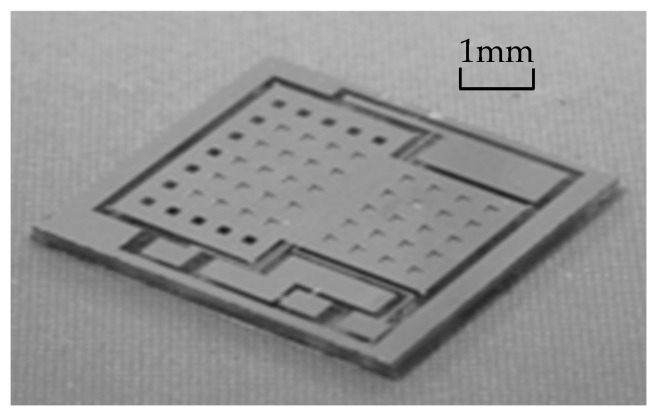
SEM image of the pendulum accelerometer.

The MEMS pendulum accelerometer has been processed at the microsystem center of Chongqing University (Chongqing, China) which consists of bulk silicon MEMS technology based on double-sided polished N-type (100 direction) silicon wafers, which have high tensile strength and low mechanical losses. The fabrication of the MEMS pendulum accelerometer includes both silicon processing and glass processing, as shown in Figure 9. First, groove windows are etched into silicon using wet etching (a). Then, the glass process forms the electrodes on the glass surface (b), followed by anode bonding of the silicon and glass (c), after which the silicon is thinned by KOH etching (d). Finally, ICP structure release is performed to create beams (e). These processes produce the sensitive unit of the pendulum accelerometer; a sample pendulum accelerometer is shown in Figure 10.

### 3.4. Interface Circuit

**Figure 11:**
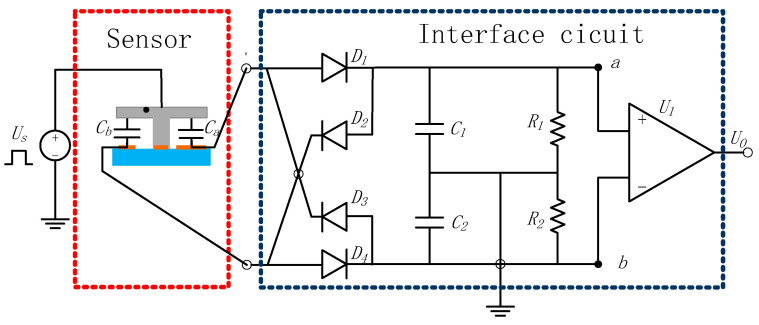
Interface circuit diagram of the pendulum accelerometer.

The interface circuit includes a ring diode capacitor voltage conversion circuit and an instrument amplifier, as shown in Figure 11. Here, *V_m_* is the voltage amplitude of the signal source *U_s_*; *C_a_* and *C_b_* are the differential capacitances of the sensor; *U_D_* comprises the voltage drops of the four ring diodes *D*_1_, *D*_2_, *D*_3_, and *D*_4_; *C*_1_ and *C*_2_ are the demodulation capacitances, where *C*_1_ = *C*_2_ = *C_L_*; *R*_1_ and *R*_2_ are the bias resistors, and *R*_1_ = *R*_2_ = *R*; *U_a_* and *U_b_* are the voltages of point *a* and *b*, respectively; and *G* is the voltage gain of the instrumentation amplifier.

According to theory [17], during a carrier cycle, when the voltage reaches a stable state during the *C*_1_ charging and discharging process, the following is true (Equation (8)):

(8) $$(2V_{m}-2U_{D}-U_{a}-∆U_{a}+U_{b}-∆U_{b})C_{a}-U_{a}T/2R_{1}=2∆U_{a}C_{1}$$

where *U_D_* is the voltage drop of diode, Δ*U_a_* and Δ*U_b_* are the voltage variation of point a and b, respectively.

Similarly, when the voltage enters a stable state during *C*_2_ charging and discharging, the following is true (Equation (9)):

(9) $$(2V_{m}-2U_{D}-U_{b}-∆U_{b}+U_{a}-∆U_{a})C_{b}-U_{b}T/2R_{2}=2∆U_{b}C_{2}$$

Finally, the output voltage is obtained as Equation (10), based on the symmetry of the circuit.

(10) $$U_{0}=G\left(U_{a}-U_{b}\right)=\frac{C_{L}}{C_{a}+C_{L}}\frac{4G\left(C_{a}-C_{b}\right)}{2\left(C_{a}+C_{b}\right)+T/R}(V_{m}-U_{D})$$

**Figure 12:**
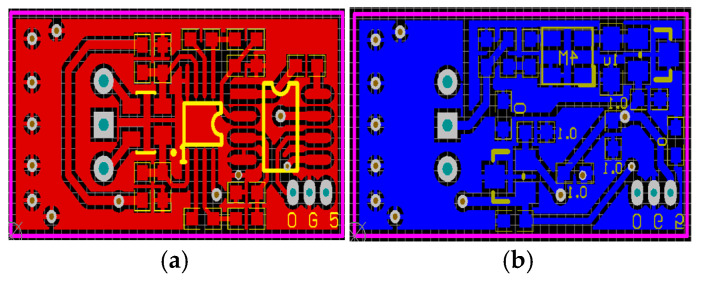
PCB design of interface circuits: (**a**) top; (**b**) back.

The interface circuits were designed as four-layer PCBs, shown in Figure 12.

## 4. Tests and Results

**Figure 13:**
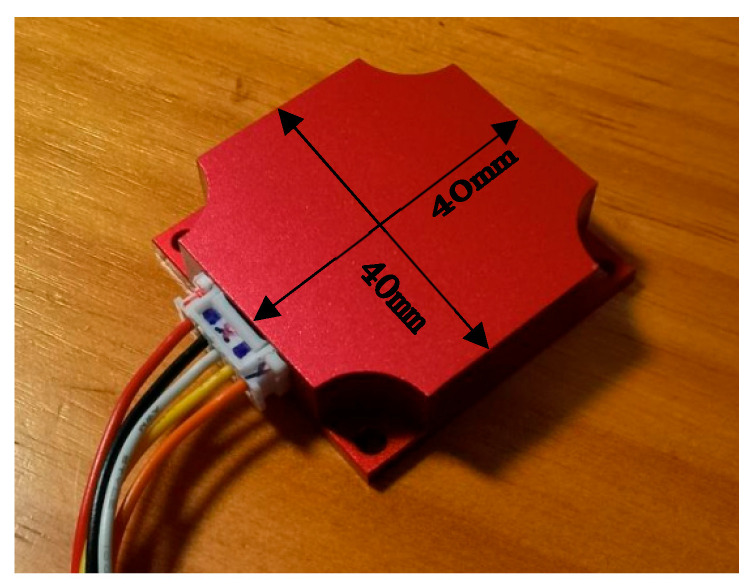
Packaged pendulum accelerometer.

The sensitive structure unit and interface circuits were integrated into a designed water-and-moisture-proof box, as shown in Figure 13. The working voltage of the accelerometer was supplied by a single power supply. The experimental platform consisted of a high-precision DC power supply, a digital multimeter, a mirror display precision rotating indexing head, and other experimental instruments [18].

**Figure 14:**
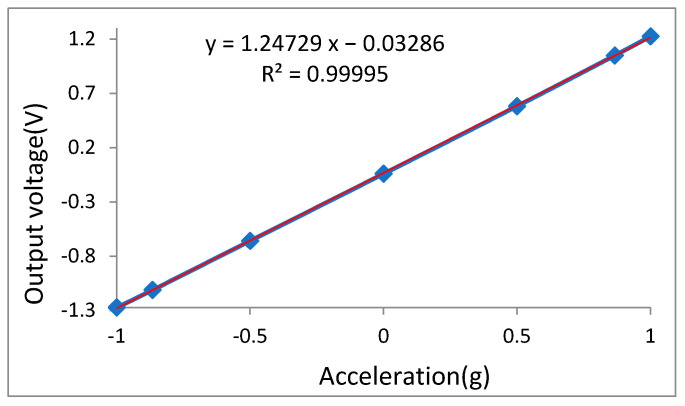
Fitted line of input–output relationship for accelerometer.

A gravitational field static rollover test was conducted to test the sensitivity, linearity, and zero-stability use of the mirroring precision rotary indexing head of Jena 1523 of the Carlzeiss (Oberkochen, Baden-Württemberg, Germany). The testing data are shown in Table 4 and Figure 14. The test results show that the measuring range is −2 g~2 g, the sensitivity is 1.247 V/g, the least-squares fitting correlation coefficient reaches 0.99995, and the nonlinearity is 0.8%.

**Figure 15:**
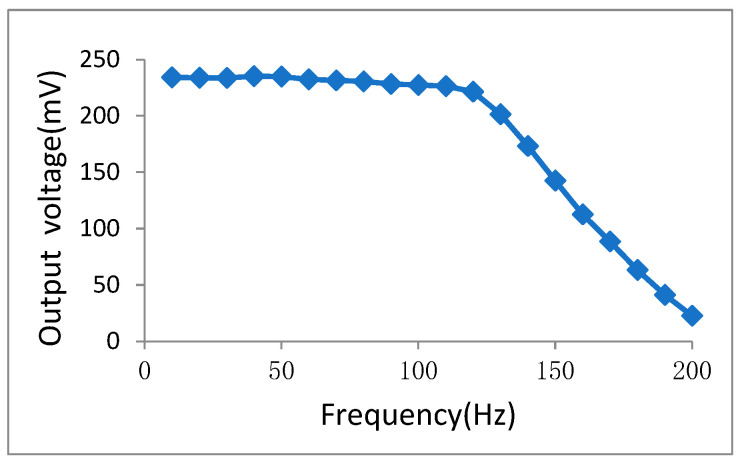
Frequency response curve of the pendulum accelerometer.

The accelerometer was mounted on a vibration table of QZ-50PP of the KINGJO (dongguan, Guangdong, China) with the vibration acceleration amplitude maintained at a constant level of 0.2 g. The excitation frequency was varied incrementally from 10 Hz in steps of 10 Hz, and the corresponding output signal amplitude of the sensor was recorded at each frequency point. The frequency response curve depicting the relationship between the system’s output signal amplitude and the input frequency was plotted, as shown in Figure 15. The −3 dB response bandwidth is determined to be approximately 143 Hz.

**Figure 16:**
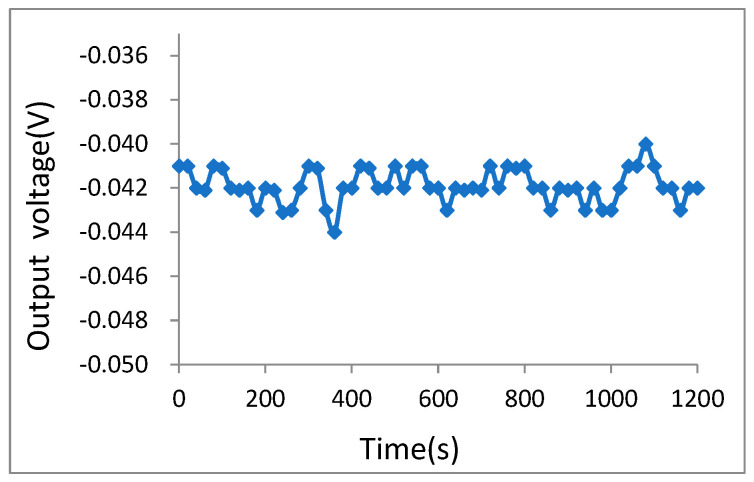
Zero-bias stability curve over 20 min.

The output voltage data were recorded continuously for 20 min, collecting data every 20 s, and the zero-bias stability curves shown in Figure 16 were plotted.

According to Equation (11) [19], the zero-bias stability of the accelerometer is calculated to be 0.610 mg.

(11) $$S_{z}=\frac{\sigma }{S_{0}}=\frac{7.60806\times 10^{-4}}{1.24729}=0.610\;mg$$

Furthermore, according to Equation (12) [20], the zero-bias stability of Allan deviation is calculated to be 0.433 μg, with the sample length 60 points, a group data length of 3 points, and 20 groups.

(12) $${S\prime }_{z}=\frac{\sqrt{\frac{1}{\left(N-1\right)}{\sum_{i=1}^{N-1}[{\bar{x}}_{i+1}\left(t\right)-{\bar{x}}_{i}\left(t\right)]}^{2}}}{1.24729}=0.433\;\mu g$$

where N is the number of groups, $\bar{x}$ is the mean value of every group.

Comparing the parameter indicators of the proposed accelerometer with those of ADXL203 of the Analog Devices (Norwood, MA, USA), as shown in Table 5, it can be seen that the sensitivity, nonlinearity, and zero-bias stability of the pendulum accelerometer developed in this study are superior to those of the ADXL203 accelerometer.

## 5. Conclusions

In this study, a novel asymmetric high-performance MEMS pendulum capacitive accelerometer is proposed. The structure and working principles of the sensor are studied in detail, and the mechanistic characteristics of its sensitive structure are analyzed using finite element analysis. The fabrication process and interface circuits are also described.

Due to the optimized design of the structure and process, it is critical that the interface circuit for the pendulum capacitive accelerometers greatly improves the system’s signal-to-noise ratio. The results of testing the pendulum accelerometer show that its sensitivity is about 1.247 V/g, and its nonlinearity is about 0.8% over a range of −2 g to 2 g. This sensor was applied to stress detection in key parts of a single-unit combination electronic truck scale to determine its vibration and balance state.

Some problems remain to be resolved; for example, performance could be significantly improved if the interface circuit were designed with ASIC technology, allowing for hybrid integration of the sensitive structure and ASIC on one chip.

## Figures and Tables

**Table 1:** Structural parameter values of the pendulum accelerometer.

Parameters	Design Value
Mass thickness	100 μm
Mass 1 length	5000 μm
Mass 1 width	2100 μm
Mass 2 length	3000 μm
Mass 2 width	2600 μm
Beam length	1100 μm
Beam width	30 μm

**Table 2:** Basic material properties of the pendulum accelerometer.

Material	Density (kg/m^3^)	Young’s Modulus (GPa)	Poisson’s Ratio
single-crystal silicon	2320	160	0.22

**Table 3:** Mode analysis results.

SET	1	2	3	4	5	6
Frequency/Hz	566.46	1967	2253.5	5778.5	9778.3	31,345

**Table 4:** Results of static gravitational field roll test.

Degree (°)	Acceleration (g)	Measure Value (V)	Fitting Value (V)	Deviation Value (V)	Nonlinearity
0	1	1.225	1.216	0.009	0.7%
30	0.866	1.048	1.049	0.001	0.08%
60	0.5	0.582	0.592	0.01	0.8%
90	0	−0.041	−0.032	0.009	0.7%
120	−0.5	−0.659	−0.656	0.003	0.24%
150	−0.866	−1.112	−1.113	0.001	0.08%
180	−1	−1.273	−1.28	0.007	0.56%

**Table 5:** Performance comparison with ADXL203 accelerometer.

Parameter	Paper	ADXL203
measure range	±2 g	±1.7 g, ±5 g, or ±18 g
sensitivity	1.248 V/g	1 V/g (normal)
nonlinearity	0.8%	1.25%
zero-bias stability	0.610 mg	25 mg

## Data Availability

The original contributions presented in this study are included in the article. Further inquiries can be directed to the corresponding author.
